# HABIT Efficacy Trial Intervention Improves Elements of General and Disease-Specific Quality of Life in Youth With Sickle Cell Disease

**DOI:** 10.1002/pbc.31990

**Published:** 2025-08-26

**Authors:** Arlene Smaldone, Deepa Manwani, Banu Aygun, Abena Appiah-Kubi, Kim Smith-Whitley, Nancy S. Green

**Affiliations:** 1 Columbia University School of Nursing, New York, New York, USA; 2 College of Dental Medicine, Columbia University Medical Center, New York, New York, USA; 3 Division of Hematology, Oncology and Cellular Therapy, Department of Pediatrics, Albert Einstein College of Medicine, New York, New York, USA; 4 Northwell, New Hyde Park, New York, USA; 5 Pediatric Hematology Oncology and Stem Cell Transplantation, Cohen Children’s Medical Center, New Hyde Park, New York, USA; 6 Division of Hematology, Children’s Hospital of Philadelphia, Philadelphia, Pennsylvania, USA; 7 Division of Pediatric Hematology, Oncology and Stem Cell Therapy, Department of Pediatrics, Columbia University Medical Center, New York, New York, USA

**Keywords:** adherence, community health worker, hydroxyurea, quality of life, sickle cell disease, text messages

## Abstract

**Background:**

Whether interventions to improve hydroxyurea adherence in youth with sickle cell disease (SCD) also improve health-related quality of life (HRQoL) has not been determined. We prospectively examined changes in generic and disease-specific HRQoL over a 12-month period in youth who participated in “Hydroxyurea Adherence for Personal Best in Sickle Cell Treatment (HABIT)” randomized controlled multi-site efficacy trial. The HABIT intervention was led by community health workers and augmented by tailored text message reminders.

**Methods:**

Improvements in generic and disease-specific HRQoL were secondary HABIT outcomes. Intervention efficacy and sustainability were measured as changes in HRQoL from Months 0 to 9 and from Months 9 to 12 of the trial, respectively. Data were analyzed for within-group and between-group changes.

**Results:**

Fifty youth, 24 assigned to intervention and 26 to the control group, mean age of 13.3 ± 1.9 years, participated in the trial. There were no differences between groups at study entry. At Month 0, total generic and disease-specific HRQoL scores were 71.2 ± 15.6 and 62.7 ± 19.0, respectively. At 9 months, compared to controls, the intervention group significantly improved generic HRQoL total score (*p* = 0.04) and two subscales (emotional, *p* = 0.03, and social, *p* = 0.01), and one disease-specific HRQoL subscale (treatment, *p* = 0.006). HRQoL improvements were not sustained at 12 months.

**Conclusions:**

Findings of this study contribute to the evolving understanding of the impact of interventions to improve hydroxyurea adherence on HRQoL in youth with SCD. Further research directed to enhancing intervention sustainability is needed to maintain hydroxyurea adherence and HRQoL improvements to reduce health disparities for youth with SCD.

## 1 | Introduction

Sickle cell disease (SCD), an inherited blood disorder with serious acute and chronic complications, affects approximately 100,000 individuals in the United States; 40% of those affected are children [1, 2]. In 2014, an expert panel convened by the National Heart, Lung, and Blood Institute recommended that most children with the generally more severe forms of SCD, HbSS and HbS-B^0^ thalassemia, age 9 months of age or older be offered treatment with hydroxyurea, a disease-modifying therapy that, among other potent effects, induces levels of fetal hemoglobin (HbF) in almost every child or adolescent [3, 4]. Use of hydroxyurea has numerous disease-related health benefits; for example, reduced pain crises and hospitalizations [5, 6], which may improve health-related quality of life (HRQoL) in children and adolescents. A recent systematic review and meta-analysis of 45 studies [7] found hydroxyurea to be effective also in preventing multiorgan damage. Despite these short- and long-term benefits, prescription of hydroxyurea to children with SCD remains low [8, 9], and daily adherence by those treated with hydroxyurea is often suboptimal [10–12].

Across chronic health conditions, youth with chronic illness report lower HRQoL compared to healthy controls [13, 14]. For those with SCD, social vulnerability [15] and racism [16, 17] may accentuate poor HRQoL. Among youth with SCD, reported HRQoL is higher for those prescribed hydroxyurea [18], adherent to its daily use [19], and have higher levels of parent support [20]. A recent systematic review found that youth with greater adherence to hydroxyurea had better physical function, emotions, social functioning, and peer relationships [19]. Of approximately 2000 respondents from 16 countries who participated in the Sickle Cell World Assessment Survey, 39% were youth 18 years or younger. Youth participants reported that SCD had the greatest negative impact on their emotional well-being and success at school [21]. Interventions to improve hydroxyurea adherence have the potential to improve HRQoL of youth with SCD.

With advances in disease-modifying therapeutic options and improved lifespan, most youth with SCD in the United States transition to adult care; therefore, improvement in patient-reported outcomes such as HRQoL of youth with SCD has become a priority [22]. Most research establishing the relationship between hydroxyurea adherence and HRQoL has been of cross-sectional design [19], and responsiveness to change is infrequently measured when testing self-management interventions to improve adherence [23]. Our earlier randomized controlled feasibility study of an intervention, led by community health workers (CHW) and augmented by tailored text message reminders, found the intervention to be feasible, acceptable to participants and results suggested improvements measured by both fetal hemoglobin (HbF) levels and hydroxyurea prescription refill data [24], the primary study outcomes, and in select domains of generic and disease-specific HRQoL, the secondary outcomes [25]. The purpose of this study was to examine secondary trial outcomes of youth self-reported changes in generic and disease-specific HRQoL over 1 year in the sample of youth and caregiver dyads who participated in the randomized controlled multisite “Hydroxyurea Adherence for Personal Best in Sickle Cell Treatment (HABIT)” efficacy trial.

## 2 | Methods

Details of the study design [26], primary outcome results [27], and youth and caregiver perspectives regarding trial participation [28] have been previously reported. The study was a four-site, 12-month 1:1 randomized controlled trial to test the efficacy and sustainability of a CHW-led intervention to improve hydroxyurea adherence among youth with SCD (HbSS or HbS-B^0^ thalassemia) ages 10–18 years. Dyad and investigator blinding were not possible; however, blinding was maintained for data analysis procedures. Youth caregiver dyads randomized to the intervention received five CHW scheduled visits over 3 months, followed by 3 months of daily text message reminders for hydroxyurea use. CHWs also accompanied the dyad to one of their clinic visits. Both groups received usual care at the clinic as well as educational handouts about SCD and hydroxyurea during the 12-month trial. Youth and caregivers completed electronic surveys either in English or Spanish at each clinic visit during the trial through a trial-specific REDCap system (http://project-redcap.org). Following the onset of the COVID-19 pandemic, CHWs’ in-person visits were shifted to virtual visits. During the period of clinic interruption, dyads completed surveys at home via emailed links. Institutional board approval was obtained at each study site before study initiation.

### 2.1 | Study Participants

English and Spanish-speaking dyads were recruited from September 2018 through December 2020 from four pediatric SCD programs at academic medical centers serving a diverse population in the New York City (three sites) and Philadelphia (one site) areas. Potentially eligible dyads were identified by medical record review of history of hydroxyurea therapy for at least 18 months, the youth’s Personal Best HbF [4] (PB-HbF), the highest recorded HbF following hydroxyurea induction, compared to the mean of two HbF levels obtained during the prior year when the youth was in a clinically steady state. An HbF decline of ≥15% from PB-HbF with hydroxyurea dosing within 5% mg/kg of dose at PB-HbF met criteria for study eligibility. To ensure eligibility at trial enrollment, an HbF value ≥15% below PB-HbF was confirmed at the initial study visit.

### 2.2 | Outcome Measures

Secondary outcomes of the HABIT Efficacy trial were generic and disease-specific HRQoL. Youth and parents completed these surveys at trial Months 0, 4, 9, and 12. Intervention efficacy and sustainability of these outcomes were measured as changes that occurred from Month 0 to Month 9 and from Month 9 to Month 12, respectively.

Generic HRQoL was measured using the PedsQL Generic Core Scale, a widely used scale with excellent psychometric properties [29] to measure HRQoL in both healthy children and those with chronic illness. The instrument consists of 23 items with four subscales (physical, emotional, social, and school) and is available in English and Spanish. Disease-specific HRQoL was measured using the PedsQL Sickle Cell Disease module [30]. Developed in 2012, the scale contains 43 items comprising nine subscales and is available in English and Spanish. Scores for both instruments range between 0 and 100, with higher scores indicating better HRQoL and are responsive to clinical change over time.

### 2.3 | Statistical Analysis

We computed Cronbach’s alpha for internal reliability in this sample as well as total and subscale scores for the intervention and control groups at each measurement timepoint. An alpha of 0.70 or higher was considered adequate reliability [31]. To assess the clinical relevance of score changes, we assessed the minimal clinically important difference (MCID) [32, 33], defined as the smallest score difference that patients perceive as beneficial [34], for each HRQoL scale and subscale. In addition, we categorized the proportion of youth with a total generic and disease-specific HRQoL score of less than 60, 60 to less than 80, or ≥80 at each timepoint as having impaired, intermediate, and good HRQoL, respectively [35].

To examine the efficacy and sustainability of the intervention on HRQoL, we examined change from baseline total and subscale scores using the MCID as a clinically meaningful difference. We then compared within- and between-group change in scores from 0 to 9 months (efficacy) and from 0 to 12 months (sustainability) using paired *t*-tests (within-group change) and Student’s *t*-tests (between-group change). We also analyzed the proportion of youth by level of HRQoL (impaired, intermediate, good) at study entry, 9 months, and 12 months using chi-square and Fisher’s exact tests. Data were analyzed using SAS 9.4 (Cary, NC) statistical software.

## 3 | Results

Results of the trial primary outcomes, improvement in HbF levels, and proportion of days covered (PDC) by hydroxyurea obtained from prescription refill data, as well as a full description of caregiver and youth participant characteristics, have been previously reported [27]. Of 83 youth who met eligibility across the four study sites, 50 youth and their caregivers participated, and 45 dyads completed the trial; 24 dyads were randomly assigned to the intervention group and 26 dyads to the control group. Youth were, on average, 13.3 ± 1.9 years old at study entry, with no differences by study group in either demographic or laboratory values. During the year prior to the trial, 60% of the youth had received acute SCD-related care in an emergency department setting, and 48% had been hospitalized. Most caregivers (64%) were employed full-time, and 50% reported being a single parent. There were no differences in youth healthcare utilization or caregiver characteristics between study groups. Study attrition was 10%; of these, three were control and two were intervention dyads. Compared to pre-enrollment levels, HbF and PDC significantly improved at 6 months within the intervention group, but not for the control group. There were no differences between the intervention and control groups at 6 months (efficacy) or 12 months (sustainability) [27].

### 3.1 | HRQoL at Study Entry

Table 1 provides HRQoL scores at study entry. Both the PedsQL Generic Core scale and PedsQL Sickle Cell Disease module demonstrated good reliability in this sample, with Cronbach’s alphas of 0.84 and 0.92 for the total score and a range of 0.88–0.91 and 0.91–0.93 for the generic and disease-specific subscale scores, respectively. At study entry, 30% of youth reported generic HRQoL scores in the intermediate or impaired range, with a mean score of 71.2 ± 15.6. Of the four subscales, scores were highest for the social subscale (78.8 ± 15.7) and lowest for the school subscale (62.1 ± 18.7). Across generic HRQoL domains, MCIDs ranged between 5.0 (social subscale) to 6.8 (physical subscale). Disease-specific HRQoL total scores, on average, were 62.7 ± 19.0, with 22% of youth reporting disease-specific total scores in the good range. Of the nine subscales, scores were highest for the Worry 2 subscale (82.0 ± 21.3) and lowest for the pain impact subscale (54.0 ± 25.4). Across the disease-specific HRQoL domains, MCIDs ranged between 5.3 (treatment subscale) and 7.7 (Communication 2 subscale) points. There were no differences in HRQoL scores between the intervention and control groups at study entry.

### 3.2 | HRQoL Improvement at 9 Months (Efficacy)

Table 2 provides the change in HRQoL scores with their respective 95% confidence intervals (CI) from study entry to 9 months. On average, generic and disease-specific HRQoL scores improved for both the intervention and control groups. For the intervention group, the generic HRQoL total score increased by 10.3 ± 12.6 points, while subscale scores increased from 5.5 ± 14.7 (physical subscale) to 16.6 ± 21.0 (emotional subscale) points above study baseline scores. Within the intervention group, generic HRQoL scores significantly increased at 9 months and exceeded MCIDs for all but the physical subscale. For the control group, the generic HRQoL scores at 9 months did not demonstrate significant within-group improvement. At 9 months, compared to the control group, the intervention group’s generic HRQoL scores significantly improved for both the total score (*p* = 0.04) and its emotional (*p* = 0.03) and social (*p* = 0.01) subscales.

At 9 months, disease-specific HRQoL for the intervention group total score increased by 16.4 ± 23.3 points, and subscale score improvement ranged between 10.2 ± 25.2 (Worry 2 subscale) and 19.9 ± 32.6 (pain impact subscale) points. Within that group, HRQoL scores significantly increased and exceeded MCIDs for all but the Worry 2 and emotions subscales. For the control group, the disease-specific HRQoL total score increased by 9.0 ± 12.0 points, and there were significant within-group HRQoL score improvements in three subscales: pain hurt (11.7 ± 20.4 increase), pain impact (13.3 ± 21.7 increase), and Worry 1 (10.0 ± 12.6 increase). At 9 months, compared to the control group, the intervention group had significantly greater improvement in the disease-specific HRQoL treatment subscale score (*p* = 0.006).

### 3.3 | HRQoL Sustained Improvement at 12 Months

Table 3 provides the changes in HRQoL scores and their respective 95% CI from study entry to 12 months. While score improvement was, on average, below that at 9 months, the intervention group demonstrated significant within-group improvement in generic HRQoL at 12 months. Score improvement exceeded MCIDs for all but the physical and emotional subscales. Similar to 9-month scores, the control group did not significantly improve generic HRQoL scores. At 12 months, there were no between-group differences in generic HRQoL scores.

At 12 months, the intervention group’s disease-specific HRQoL total score increased by 12.2 ± 19.3 points, and subscale score improvement ranged between 4.8 ± 32.7 (emotions) and 17.1 ± 30.5 (Communication 1) points. Within-group HRQoL scores significantly increased and exceeded MCIDs for total score and pain hurt, pain impact, Worry 1, and Communication 1 subscales. For the control group, significant within-group increases in disease-specific HRQoL were seen for total score (8.3 ± 16.2 increase) and two subscales: pain impact (13.2 ± 21.9 increase) and emotions (11.4 ± 24.1 increase). At 12 months, compared to the control group, there were no differences in disease-specific HRQoL scores.

**FIGURE 1 |:**
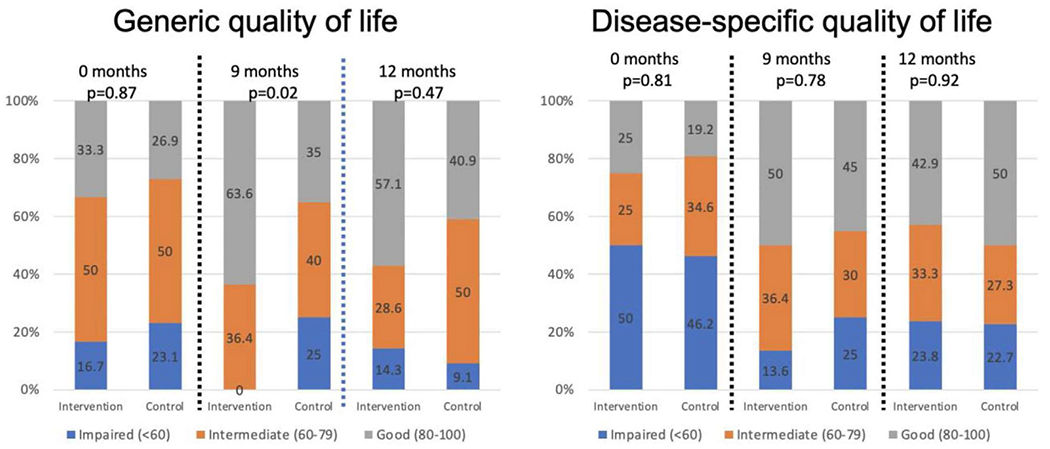
Categorized generic and disease-specific sickle cell quality of life at 0, 9, and 12 trial months. *Note:* Generic quality of life measured using the PedsQL Generic Core Scale; disease-specific quality of life measured using the PedsQL Sickle Cell Disease module.

Figure 1 displays total and disease-specific HRQoL scores at study entry, 9, and 12 months, categorized as good, intermediate, and impaired. At 9 months, more intervention youth had generic HRQoL scores in the “good” range compared to the control group (*p* = 0.02). There were no differences between groups for disease-specific HRQoL at 9 months or generic and disease-specific HRQoL at 12 months.

## 4 | Discussion

We assessed generic and disease-specific HRQoL of youth who participated in a 12-month multi-site randomized controlled trial to test the efficacy and sustainability of a CHW-led intervention to improve hydroxyurea adherence. At 9 months, compared to the control group, the intervention group significantly improved generic (total score and emotional and social subscales) and disease-specific (treatment subscale) HRQoL scores beyond a minimal clinically important difference. At 12 months, these improvements were not sustained. Similarly, when total scores were categorized into ranges reflecting good, intermediate, and impaired HRQoL, at 9 months, more youth in the intervention group had generic total scores reflecting good HRQoL compared to the controls. However, there were no between-group differences in disease-specific HRQoL total scores at either 9 or 12 months or in generic HRQoL total scores at 12 months. These findings suggest that while the intervention was efficacious in improving some domains of HRQoL, the intervention effect was not sustainable. These findings are consistent with those of our earlier two-site HABIT feasibility trial, which demonstrated efficacy in similar HRQoL domains at 6 months [25].

The HABIT Efficacy trial was designed to improve adherence to hydroxyurea, the primary outcome. However, improvement in youth self-reported HRQoL was not consistent with adherence measured by HbF levels and proportion of days covered (PDC) by hydroxyurea. While adherence at 6 months significantly improved for the HABIT intervention group, there were no significant differences when compared to the control group [27]. This suggests that the intervention had a greater effect on HRQoL, particularly for generic HRQoL domains, than it did on hydroxyurea adherence itself. It is also possible that behavior change required for hydroxyurea adherence to improve HbF levels and PDC may take longer to achieve and/or require a more intensive intervention.

Most prior studies examining the relationship between HRQoL and hydroxyurea adherence have been of cross-sectional design [19]. While it is clear that the youth prescribed hydroxyurea have higher HRQoL compared to those not prescribed hydroxyurea [19], few clinical trials designed to improve hydroxyurea adherence have examined the effect of the intervention on HRQoL. Findings of our study, as well as one other recent clinical trial, have begun to address this gap; however, results are mixed. The Start Healing in Patients with Hydroxyurea (SHIP-HU) [36, 37] study randomized youth and young adults (median age 26.4 years) to a CHW case management intervention plus subspecialty care or to a control group who received subspecialty care alone. The support provided by CHWs was separate and distinct from the clinical care that the intervention group received. Contrary to our study findings, there were no differences between study groups in either hydroxyurea adherence, the primary outcome, or HRQoL, a secondary outcome. These differing findings may, in part, be due to participant age differences and the HABIT trial’s strategy of enrolling youth–caregiver dyads. Caregiver participation with their youth may have provided additional support toward promoting youth adherence. Moreover, HABIT CHWs coached the dyads to jointly track hydroxyurea adherence through prospective youth HbF values, which may be a more tangible measure of hydroxyurea adherence.

Systematic reviews have demonstrated that CHWs are effective in improving chronic illness self-management in adults [38] and youth [39] and in reducing preventable healthcare utilization [40]. In addition, CHWs can be a cost-effective adjunct to care, especially among low-resourced, underserved communities [38, 41, 42]. Of note, none of these reviews or economic evaluations included youth or adults with SCD. Hsu and colleagues [43] reviewed US-based and global projects employing CHWs as adjuncts to SCD clinical care, concluding that CHW integration within standard care had the potential to improve short- and long-term outcomes, including HRQoL, and reduce health disparities. More recently, two reports from the National Academies of Science, Engineering, and Medicine [44] and the Sickle Cell Disease Association of America [45] provide strong support for the role of CHWs within SCD clinical care. Future research is needed to test the effect, sustainability, and cost-effectiveness of CHW interventions for people living with SCD.

## 5 | Summary/Conclusion

These findings must be interpreted considering study limitations. Our sample size was modest and likely underpowered to estimate the effect in several HRQoL domains, even when score changes from baseline exceeded MCID. The intervention employed both CHW visits and tailored text message reminders, precluding examination of each effect separately. Dyads were not blinded to group assignment; however, study attrition was low among both trial groups, suggesting that lack of blinding did not affect trial results. As all study sites were located within urban centers in the Northeast of the United States, findings may not be generalizable to other locations. The HABIT Efficacy trial was conducted during the COVID-19 pandemic; it is possible that the temporary disruption from usual clinical care and school attendance may have affected both hydroxyurea adherence and HRQoL. Nonetheless, findings of our study contribute to the evolving literature regarding interventions to improve HRQoL in youth with SCD.

Our findings suggest that the HABIT intervention had a positive effect in select domains of both generic and disease-specific HRQoL at 9 months, but that these effects were not sustained at 12 months. HABIT participants in the intervention group in both the feasibility and efficacy trials reported that they enjoyed working with CHWs and learned new information about both hydroxyurea and SCD [24, 28]. Further research directed to improve the sustainability of interventions is needed to improve hydroxyurea adherence and HRQoL to reduce health disparities for youth with SCD.

## Figures and Tables

**TABLE 1 |:** Generic- and disease-specific quality of life total and subscale scores and dyad concordance at study entry.

Variable	Total sample (*N* = 50) %	Alpha	MCID	Intervention (*N* = 24) %	Control (*N* = 26) %	*p*-value

*Generic quality of life*						
Total score (categorized)						0.87
% Impaired (0–59)	20	NA	NA	16.7	23.1	
% Intermediate (60-79)	50	NA	NA	50.0	50.0	
% Good (80–100)	30	NA	NA	33.3	26.9	
	Mean ± SD			Mean ± SD	Mean ± SD	
Total score	71.2 ± 15.6	0.84	6.2	73.5 ± 14.5	69.1 ± 16.6	0.32
Subscale scores						
Physical	71.8 ± 19.6	0.88	6.8	75.9 ± 16.0	68.0 ± 22.0	0.15
Emotional	71.7 ± 20.2	0.91	6.1	71.3 ± 19.5	72.1 ± 21.3	0.88
Social	78.8 ± 15.7	0.90	5.0	81.7 ± 14.5	76.2 ± 16.6	0.22
School	62.1 ± 18.7	0.88	6.5	63.8 ± 20.5	60.6 ± 17.1	0.55
*Disease-specific quality of life*						
Total score (categorized)						0.81
% Impaired (0–59)	48.0			50.0	46.2	
% Intermediate (60–79)	30.0			25.0	34.6	
% Good (80–100)	22.0			25.0	19.2	
	Mean ± SD			Mean ± SD	Mean ± SD	
Total score	62.7 ± 19.0	0.91	5.7	63.0 ± 19.2	62.5 ± 19.1	0.93
Subscale scores						
Pain hurt	64.9 ± 22.7	0.92	6.4	67.0 ± 22.6	62.9 ± 23.0	0.53
Pain impact	54.0 ± 25.4	0.91	7.6	53.6 ± 26.0	54.3 ± 25.3	0.93
Pain management	62.0 ± 23.5	0.92	6.6	59.4 ± 26.6	64.4 ± 20.5	0.45
Worry 1	67.0 ± 23.8	0.92	6.7	65.6 ± 24.6	68.3 ± 23.4	0.70
Worry 2	82.0 ± 21.3	0.93	5.6	80.7 ± 22.7	83.2 ± 20.3	0.69
Emotions	61.8 ± 31.7	0.93	8.4	62.0 ± 33.9	61.5 ± 30.2	0.96
Treatment	72.1 ± 18.6	0.92	5.3	72.5 ± 17.4	71.8 ± 19.9	0.91
Communication 1	69.5 ± 25.7	0.93	6.8	69.5 ± 26.4	69.4 ± 25.4	0.99
Communication 2	59.5 ± 27.3	0.92	7.7	61.8 ± 26.7	57.4 ± 28.2	0.57

*Note:* Generic quality of life = PedsQL Generic Core Scale. Disease-specific quality of life = PedsQL Sickle Cell Disease module.

Abbreviations: CI, confidence interval; NA, not applicable; SD, standard deviation.

**TABLE 2 |:** Within- and between-group change in youth generic and disease-specific quality of life total and subscale scores from study entry to 9 months (efficacy).

Variable	Intervention (*N* = 22)		*p*-value	Control (*N* = 20)		*p*-value
	Mean change ± SD	95% CI		Mean change ± SD	95% CI	
***Generic quality of life***						
Total score	10.3 ± 12.6	4.7, 15.6	0.001	2.0 ± 12.3	−3.8, 7.8	0.47
Subscale scores						
Physical	5.5 ± 14.7	−1.0, 12.1	0.09	0.8 ± 20.6	−8.9, 10.4	0.87
Emotional	16.6 ± 21.0	7.3, 25.9	0.001	2.3 ± 19.3	−6.8, 11.3	0.60
Social	10.2 ± 9.2	6.2, 14.3	<0.001	1.0 ± 12.9	−5.1, 7.1	0.73
School	11.6 ± 20.5	2.5, 20.7	0.01	4.8 ± 18.2	−3.8, 13.3	0.26
***Disease-specific quality of life***						
Total score	16.4 ± 23.3	6.0, 26.7	0.003	9.0 ± 12.0	3.4, 14.6	0.003
Subscale scores						
Pain hurt	12.6 ± 23.2	2.4, 22.9	0.02	11.7 ± 20.4	2.1, 21.2	0.02
Pain impact	19.9 ± 32.6	5.4, 34.3	0.009	13.3 ± 21.7	3.1, 23.4	0.01
Pain management	17.0 ± 35.7	1.2, 32.9	0.04	8.8 ± 19.9	−0.6, 18.1	0.06
Worry 1	19.8 ± 29.4	6.7, 32.8	0.005	10.0 ± 12.6	4.1, 15.9	0.002
Worry 2	10.2 ± 25.2	−0.9, 21.4	0.07	8.1 ± 19.1	−0.8, 17.1	0.07
Emotions	15.3 ± 37.8	−1.4, 32.1	0.07	9.4 ± 20.6	−0.3, 19.0	0.06
Treatment	15.6 ± 19.8	6.8, 24.3	0.001	1.4 ± 10.1	−3.3, 6.2	0.54
Communication 1	19.8 ± 32.3	5.5, 34.1	0.009	4.4 ± 24.0	−6.8, 15.7	0.42
Communication 2	18.2 ± 34.6	2.9, 33.5	0.02	9.6 ± 23.8	−1.5, 20.7	0.09

*Note: p*-values displayed within the table represent within-group change analyzed by paired *t*-tests. Generic quality of life = PedsQL Generic Core Scale. Disease-specific quality of life = PedsQL Sickle Cell Disease module. Between-group changes, analyzed via Student’s *t*-test, were present for the following generic (total score *p* = 0.04; emotional subscale *p* = 0.03, social subscale *p* = 0.01) and disease-specific (treatment subscale *p* = 0.006) subscales.

Abbreviations: CI, confidence interval; SD, standard deviation.

**TABLE 3 |:** Within- and between-group change in youth generic and disease-specific quality of life total and subscale scores from study entry to 12 months (sustainability).

Variable	Intervention (*N* = 21)		*p*-value	Control (*N* = 22)		*p*-value
	Mean change ± SD	95% CI		Mean change ± SD	95% CI	
***Generic quality of life***						
Total score	7.9 ± 14.5	1.3, 14.5	0.02	3.8 ± 13.7	−2.3, 9.9	0.21
Subscale scores						
Physical	4.2 ± 18.0	−4.0, 12.4	0.30	3.4 ± 19.7	−5.3, 12.2	0.43
Emotional	10.0 ± 24.0	−0.9, 20.9	0.07	4.3 ± 22.2	−3.5, 14.1	0.37
Social	9.8 ± 13.7	3.5, 16.0	0.004	2.7 ± 15.8	−4.3, 9.7	0.42
School	9.8 ± 18.3	1.4, 18.1	0.02	5.0 ± 16.6	−2.3, 12.4	0.17
***Disease-specific quality of life***						
Total score	12.2 ± 19.3	3.4, 20.9	0.009	8.3 ± 16.2	1.2, 15.5	0.02
Pain hurt	13.0 ± 21.6	3.1, 22.8	0.01	10.1 ± 26.5	−1.7, 21.9	0.09
Pain impact	14.9 ± 28.3	2.0, 27.8	0.03	13.2 ± 21.9	3.5, 22.9	0.01
Pain management	15.5 ± 34.7	−0.3, 31.3	0.05	11.4 ± 29.6	−1.8, 24.5	0.09
Worry 1	16.2 ± 28.2	3.3, 29.0	0.02	7.0 ± 26.1	−4.5, 18.6	0.22
Worry 2	7.7 ± 28.4	−5.2, 20.6	0.22	3.4 ± 20.1	−5.5, 12.3	0.44
Emotions	4.8 ± 32.7	−10.1, 19.7	0.51	11.4 ± 24.1	0.7, 22.0	0.04
Treatment	7.5 ± 20.3	−1.8, 16.7	0.11	4.4 ± 12.0	−0.9, 9.7	0.10
Communication 1	17.1 ± 30.5	3.2, 30.9	0.02	−1.6 ± 24.8	−12.6, 9.4	0.77
Communication 2	10.7 ± 23.3	0.1, 21.3	0.05	7.6 ± 30.9	−6.1, 21.3	0.26

*Note: p*-values displayed within the table represent within-group change analyzed by paired *t*-tests. Generic quality of life = PedsQL Generic Core Scale. Disease-specific quality of life = PedsQL Sickle Cell Disease module. Between-group changes, analyzed via Student’s *t*-test, were not present for any generic quality-of-life score; between-group changes were present for one disease-specific subscale score, Communication 1 (*p* = 0.03).

Abbreviations: CI, confidence interval: SD, standard deviation.

## Data Availability

The data that support the findings of this study are available from the corresponding author upon reasonable request.

## Abbreviations:

CHW: community health workers
CI: confidence interval
HABIT: Hydroxyurea Adherence for “Personal Best” in Sickle Cell Treatment
HbF: fetal hemoglobin
HRQoL: health-related quality of life
MCID: minimal clinically important difference
PB-HbF: personal best fetal hemoglobin
PDC: proportion of days covered
SCD: sickle cell disease
SD: standard deviation
